# Ancestral Genomes, Sex, and the Population Structure of Trypanosoma cruzi

**DOI:** 10.1371/journal.ppat.0020024

**Published:** 2006-03-31

**Authors:** Jorge M. de Freitas, Luiz Augusto-Pinto, Juliana R Pimenta, Luciana Bastos-Rodrigues, Vanessa F Gonçalves, Santuza M. R Teixeira, Egler Chiari, Ângela C. V Junqueira, Octavio Fernandes, Andréa M Macedo, Carlos Renato Machado, Sérgio D. J Pena

**Affiliations:** 1 Departamento de Bioquímica e Imunologia, Universidade Federal de Minas Gerais, Belo Horizonte, Brazil; 2 Departamento de Parasitologia, Universidade Federal de Minas Gerais, Belo Horizonte, Brazil; 3 Departamento de Medicina Tropical, Fundação Oswaldo Cruz, Rio de Janeiro, Brazil; Stanford University, United States of America

## Abstract

Acquisition of detailed knowledge of the structure and evolution of Trypanosoma cruzi populations is essential for control of Chagas disease. We profiled 75 strains of the parasite with five nuclear microsatellite loci, 24Sα RNA genes, and sequence polymorphisms in the mitochondrial cytochrome oxidase subunit II gene. We also used sequences available in GenBank for the mitochondrial genes cytochrome B and NADH dehydrogenase subunit 1. A multidimensional scaling plot (MDS) based in microsatellite data divided the parasites into four clusters corresponding to T. cruzi I (MDS-cluster A), T. cruzi II (MDS-cluster C), a third group of *T. cruzi* strains (MDS-cluster B), and hybrid strains (MDS-cluster BH). The first two clusters matched respectively mitochondrial clades A and C, while the other two belonged to mitochondrial clade B. The 24Sα rDNA and microsatellite profiling data were combined into multilocus genotypes that were analyzed by the haplotype reconstruction program PHASE. We identified 141 haplotypes that were clearly distributed into three haplogroups (X, Y, and Z). All strains belonging to T. cruzi I (MDS-cluster A) were Z/Z, the T. cruzi II strains (MDS-cluster C) were Y/Y, and those belonging to MDS-cluster B (unclassified T. cruzi) had X/X haplogroup genotypes. The strains grouped in the MDS-cluster BH were X/Y, confirming their hybrid character. Based on these results we propose the following minimal scenario for T. cruzi evolution. In a distant past there were at a minimum three ancestral lineages that we may call, respectively, T. cruzi I, T. cruzi II, and T. cruzi III. At least two hybridization events involving T. cruzi II and T. cruzi III produced evolutionarily viable progeny. In both events, the mitochondrial recipient (as identified by the mitochondrial clade of the hybrid strains) was T. cruzi II and the mitochondrial donor was T. cruzi III.

## Synopsis

The parasite protozoan *Trypanosoma cruzi* causes Chagas disease, a malady that afflicts almost 20 million people in South America and Central America. Although the genome sequencing of T. cruzi has been recently completed, little is known about its population structure and evolution. Since 1999, two major evolutionary lineages presenting distinct epidemiological characteristics have been recognized in the parasite: T. cruzi I and T. cruzi II, the latter being much more associated with severe chronic cases of the disease. We describe new and important aspects of the population structure of the parasite, especially the characterization of a third ancestral lineage that we propose to call T. cruzi III. Through careful dissection of the genetic constitution of blocks of genes that are stably transmitted from generation to generation of the parasite we deduced at least two occurrences of the formation of hybrid strains from the parental lineages T. cruzi II and T. cruzi III, including the strain CLBrener, whose genome was sequenced. We did not find any hybrids originating from T. cruzi I. A fascinating finding was that both hybrids studied had the same mitochondrial DNA type as the T. cruzi III ancestral lineage, which was quite different from *T.cruzi* II.

## Introduction

The parasite protozoan *Trypanosoma cruzi* causes Chagas disease, a malady that afflicts almost 20 million people in South America and Central America, with more than 20,000 deaths reported each year [1,2]. Two different ecosystems exist for *T. cruzi:* one related to wild hemiptera and generally involving wild mammals (the “sylvatic” cycle), and another dependent on home-dwelling hemiptera and primarily involving humans and household animals (the so-called “domestic” cycle). The connection between the two ecosystems is made by infected rats, mice, bats, marsupials, and other feral mammals. It is estimated that the parasite emerged as a species well over 150 million years ago, originally infecting primitive mammals dispersed throughout Laurasia and Gondwanaland, regions that originated North and South America, respectively [3]. The first contact with humans occurred much more recently, in the late Pleistocene, 15,000–20,000 years ago, when humans first peopled the Americas—thus, Homo sapiens is a very recent new host for T. cruzi. There is convincing molecular evidence for the presence of T. cruzi DNA in mummies exhumed in Northern Chile and Southern Peru and dating as far back as 9,000 years before the present day. [4].

The conventional mode of transmission of T. cruzi to humans is by the feces of infected hematophagous triatomine bugs. Alternative modes of infection include blood transfusion, congenital transmission from infected mothers, and ingestion of contaminated foods. Thanks to intensive programs of triatomine control, vectorial infection has been virtually abolished in Brazil, Chile, Uruguay, and Argentina [5]. Moreover, improved screening of blood donors to reduce the likelihood of transfusional transmission and early detection and treatment of congenital cases have added to this success. It would be, however, a mistake to think that Chagas disease has been controlled. High levels of vector-borne transmission are still apparent in many areas, and several of the endemic countries have yet to develop serious large-scale surveillance and intervention programs [5]. Also, migrations of infected individuals offer a risk of new transmission in previously nonendemic regions, such as the United States [6]*.* Furthermore, the ancient and wide-ranging sylvatic cycle constitutes an enormous reservoir of parasites that represents a threat for humans.

Recent studies have shown that in a nonendemic area of the Brazilian Atlantic coastal rainforest 50% of the triatomine vectors and of the marsupials Didelphis marsupialis and Philander opossum [7] as well as 52% of the golden lion tamarins and several other species of New World primates [8] were naturally infected with T. cruzi. Moreover, in the United States T. cruzi has been found in 11.4% of opossums and 22% of the raccoons, together with infected triatomine bugs in the state of Georgia [9]. In certain areas of that state 43% of the raccoons were infected [10]. Closer to the human domestic environment, Bradley et al. [11] have shown that 3.6% of the rural hunting dogs in Oklahoma were seropositive for *T. cruzi.* Human infection from the sylvatic environment can occur either from sudden migration of hemiptera to the human environment, forced by the destruction of forests [12] or by the ingestion of foods contaminated by the feces of hemipterae or by crushed insects [13,14]. Thus, a complete understanding of the population structure of *T. cruzi,* especially the sylvatic cycle, will be indispensable for controlling the disease.

T. cruzi is diploid, with different-sized homologous chromosome pairs [15]. Its genome has been recently sequenced [16], and its size (diploid) has been estimated between 106.4 and 110.7 Mb. At least 50% of the T. cruzi genome is made up of repetitive sequences, consisting of large gene families of surface proteins, retrotransposons, and subtelomeric repeats.

There is extensive and well-characterized intraspecific genetic diversity in *T. cruzi* (reviewed in [17,18]). Two major evolutionary lineages of the parasite, named T. cruzi I and T. cruzi II, have been identified [19]. These lineages are very divergent as revealed by several biological and molecular markers, including isozymes, 24Sα rDNA, and mini-exon gene polymorphisms [20]. T. cruzi I and T. cruzi II strains belong predominantly to distinct ecological environments: respectively, the sylvatic and domestic transmission cycles of Chagas disease [3,21]. T. cruzi I strains are characterized by zymodeme Z1 (a zymodeme is a group of strains that have the same isozyme profile), 24Sα rDNA group 2, and mini-exon group 2, and induce low parasitism in human chagasic patients. In contrast, T. cruzi II strains are characterized by zymodeme Z2, 24Sα rDNA and mini-exon group 1, and cause human infections with high parasitemia in classic endemic areas [21]. At least in Brazil, T. cruzi II strains appear to be exclusively responsible for tissue lesions in Chagas disease [22]. Additionally, there are some parasite strains that cannot be properly grouped into any one of these two major lineages. Among these unclassified strains are those identified as belonging to zymodeme Z3 [23] and other hybrid strains characterized as rDNA group 1/2 [24,25]. Using isozymes and random amplified polymorphic DNA (RAPD) typing, Brisse et al. [26] proposed that T. cruzi II strains could be partitioned into five phylogenetic sublineages (IIa-e), each comprising one of the following reference strains: CanIII cl1 (IIa), Esmeraldo cl3 (IIb), M5631 cl5 (IIc), MN cl2 (IId), and CLBrener (IIe). In contrast, *T. cruzi* I strains could not be further subdivided. Within each of these clades or sublineages, there is extensive genetic diversity that can be unraveled by analyses with microsatellites and several other genomic markers (reviewed in [27]).

Although capable of recombination in vitro [28], T. cruzi reproduces predominantly by binary fission and consequently its diploid nuclear genotype is transmitted *en bloc* to the progeny. Thus, the parasite presents extreme degrees of linkage disequilibrium, as shown through isozymes [29] and microsatellites [30], and exhibits a predominantly clonal population structure. Indeed, T. cruzi still has been considered the paradigm for clonal eukaryotic pathogenic microorganisms [31]. The occurrence of hybrid strains in natural populations of T. cruzi was suggested by isozyme analyses [32,33], restriction fragment-length polymorphism (RFLP) of housekeeping genes [34], RAPD [35], and genotype variations observed at chromosomal level [15,35,36], and has been confirmed using nucleotide sequences [37,38]. Their discovery proved that sexual events definitely have taken place in the past and have shaped the genetical structure of current T. cruzi populations. However, such genetic exchange events seem to have been rare enough to allow the propagation of clonal genotypes over long periods of time and wide geographical regions [35]. Because of the linkage disequilibrium, genotyping of nuclear markers in T. cruzi has thus far been limited to characterization of multilocus genotypes. Therefore, to understand the evolutionary history of the species it would be desirable to dissect the multilocus genotypes into their constituent haploid genome blocks. We wish to report that we have achieved this, revealing the existence of ancestral haplogroups and repeated hybridization events in T. cruzi.

## Results

**Figure 1:**
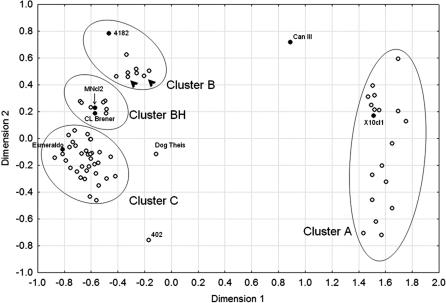
Multidimensional Scaling Plot of 75 T. cruzi Strains Genotyped for Five Microsatellites Only outliers and the prototypical strains of Brisse et al. [26] are named in the plot. Arrowheads indicate strains 222 and 115. The strains in the areas delimited by ellipsoids are the following: MDS-cluster A: 1001, 1004, 1006, 1502, 1523, A83, A87, Col18/05, Colombiana, Cuíca, Cutia, D7, Gambacl1, Rb1, Rb2, Rb6, SE, X10cl1, 402, Mas1cl1, 84, 207, 209, 239, 577, 578, 580, 581, 803, 1005, 1014, 1043, 1931, 183744, 169/1; MDS-cluster C: 200pm, 84Ti, Be62, CPI11/94, CPI95/94, Esmeraldo, Gil, GLT564, GLT593, GMS, GOCH, Ig539, JAF, JG, JHF, JSM, MPD, OPS27/94, Tu18 cl11, Y; MDS-cluster B: 115, 222, 226, 231, 3663, 3869, 4182, M5631cl5; MDS-cluster BH: M6241cl6, 167, 1022, c182, CLBrener, MNcl2, NR, SC43 cl1, SO3, Tulacl2.

We have typed 75 strains of T. cruzi (Table 1) with five nuclear CA-repeat microsatellites (Table S1). We assumed a stepwise mutation model for the evolution of microsatellites and used the minimum number of mutational steps necessary to transform one strain microsatellite profile into another to build a genetic distance matrix. The multidimensional scaling (MDS) plot shown in Figure 1 provided, with excellent fit (stress = 0.08), a visual representation of the distance matrix. Four clusters are clearly visible and identified by ellipsoids in the MDS plot. The identity of the clusters is revealed by the presence of the prototypical strains of Brisse et al. [26]: MDS-cluster A corresponds to T. cruzi I, MDS-cluster C to T. cruzi IIb, MDS-cluster B to T. cruzi IIc, and MDS-cluster BH to the IId and IIe sublineages. Only three strains fell outside the four clusters: CanIII (sub-lineage IIa), Dog Theis, and 402.

**Table 1:**
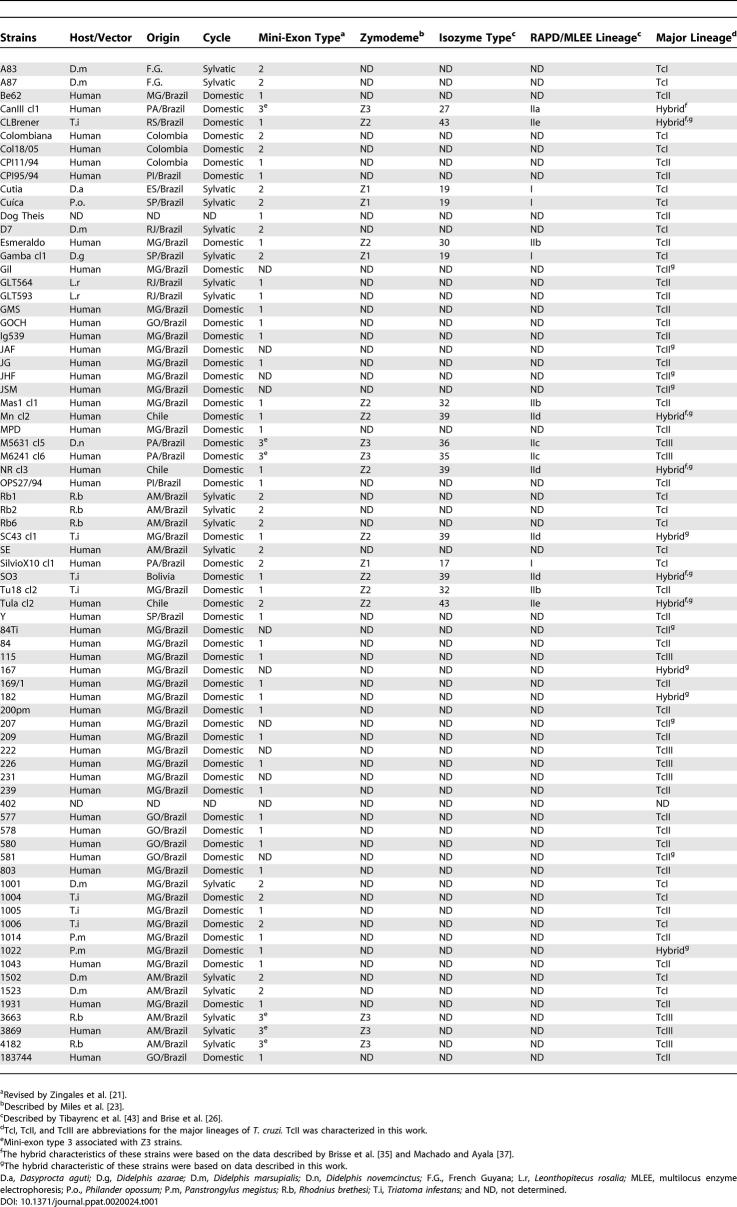
Characteristics of T. cruzi Strains Analyzed

**Figure 2:**
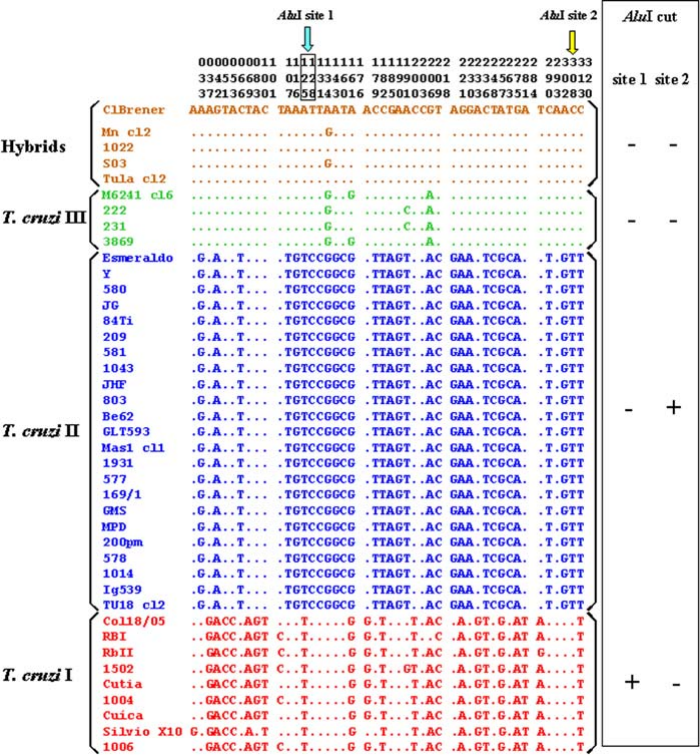
Sequence of a 290–Base Pair Fragment of the Mitochondrial *COII* Gene of 41 Strains of T. cruzi Only the variable nucleotides are shown. T. cruzi III refers to sublineage IIc, and “Hybrids” indicate the strains belonging to sublineages IId and IIe of Brisse et al [26]. Two AluI restriction sites are indicated. RFLP analysis of these two sites allows unambiguous classification of T. cruzi strains to the three mitochondrial clades as shown on the right hand side.

**Figure 3:**
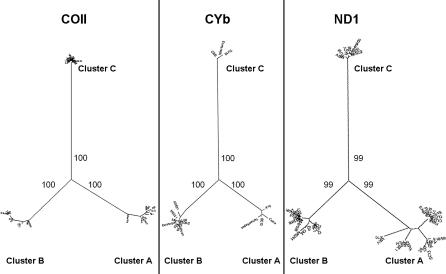
NJ Trees Obtained from the Sequences of Three Mitochondrial Genes of *T. cruzi: COII, CYb,* and *ND1* The numbers in the three main branches indicate the percentage bootstrap values. For *COII* the strains the following clades are displayed: clade A: 1004, 1006, 1502, Col18/05, Cuica, Cutia, RBI, RbII, SilvioX10; clade B: sublineage IIc—222, 231, 3869, M6241, Mn, sublineages IId and IIe—CLBrener, 1022, SO3, Tula; clade C: 1014, 1043, 169/1, 1931, 200pm, 209, 577, 578, 580, 581, 803, 84Ti, Be62, Esmeraldo, GLT593, GMS, Ig539, JG, JHF, Mas1, MPD, TU18, Y. For *CYb* the strains are as follows: clade A: Cuica, SC13, Tehuentepec, X10; clade B: sublineage IIc—M5631, M6241, X109/2, sublineages IId and IIe—92.80, CL, Guateque, MN, SC43, Tulahuen, X57; clade C: CBB, Esmeraldo, TU18. For *ND1* the strains are as follows: clade A: 133, 26, 85/818, A80, A92, CEPA, CUICA, CUTIA, Esquilo, MAV, OPS21, P0AC, P209, SABP3, SC13, SO34, TEH, V121, Vin, X10; clade B: sublineage IIc—CM, M6241, X109/2, X110/8, X9/3, sublineages IId and IIe—CL, 86/2036, 86–1, EPP, P251, P63, PSC-O, SO3, Tulahuen, VMV4; clade C: CBB, Esmeraldo, MBV, MCV, MSC2, TU18, X-300.

In 41 of the 75 strains we sequenced a 290–base pair region of the maxicircle-encoded cytochrome oxidase subunit II *(COII)* gene encompassing 44 variable positions (Figure 2). The sequenced data were used to generate a neighbor-joining (NJ) tree that is shown in Figure 3. It is clear that there are three tightly clustered sets of strains, separated by very large genetic distances, permitting straightforward allocation of T. cruzi strains into three mitochondrial clades that can also be simply identified by variation in just two AluI RFLP sites (Figure 2), which were then scored for all 75 strains (Table 2). Our MDS clusters corresponded perfectly to these mitochondrial clades, with the exceptions of MDS-clusters B (sublineage IIc) and BH (thus called because it contains the hybrid sublineages IId and IIe), both of which fall within mitochondrial clade B. To confirm our finding, we also built NJ trees for sequences obtained from GenBank of two other mitochondrial genes, cytochrome b *(CYb)* [35] and NADH dehydrogenase subunit 1 *(ND1)* [37]. The *CYb* and *ND1* trees had very similar topology to that of the *COII* tree (all with extremely high bootstrap values for the three main branches), confirming that sublineages IIc, IId, and IIe indeed belong to the same mitochondrial clade (Figure 3). We tested this notion further using analysis of molecular variance [39]. By partitioning the variability within and between mitochondrial clades we found that for *COII, CYb,* and *ND1,* respectively, 97%, 91%, and 68% of the genetic variability was found among clades.

**Table 2:**
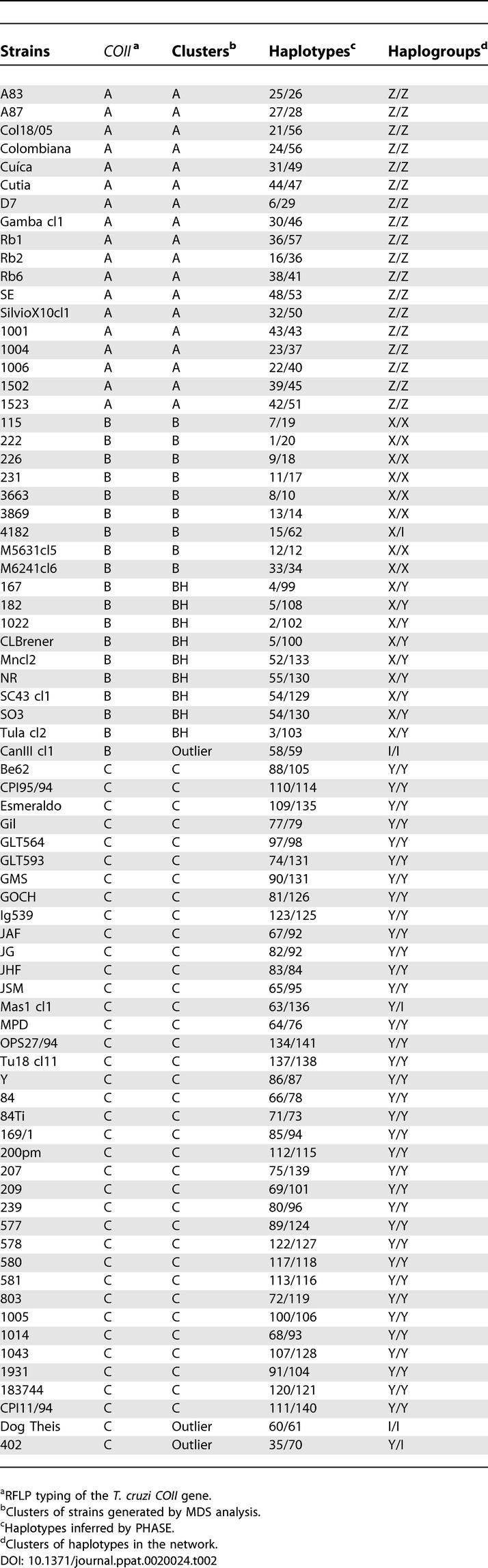
Nuclear and Mitochondrial Markers of T. cruzi Inferred in This Work

**Figure 4:**
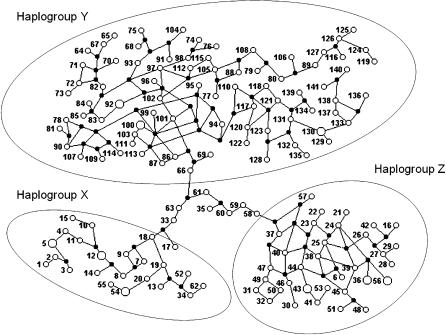
Median Joining Network of the Haplotypes Identified by the PHASE Software

We also typed all strains for the polymorphism of the D7 divergent domain of the 24Sα rRNA gene (Table S1) and combined the results with the microsatellites into multilocus genotypes that were analyzed with the PHASE software [40]. We identified 141 different haplotypes corresponding to a haplotypic diversity of 0.993. The identified haplotypes were then subjected to a median joining analysis using the NETWORK 3.1 software [41]. The resulting multitude of plausible trees is best expressed by a network that displays alternative potential evolutionary paths (Figure 4). Three haplotypic clusters are clearly identifiable: we called them haplogroups X, Y, and Z. Within these haplotypic clusters there is extensive reticulation because of the stepwise recurrent nature of microsatellite mutations [42]. However, the three haplogroups are connected by long and unique paths, emphasizing the great genetic distance between them. Seven haplotypes (numbers 33, 35, 58, 59, 60, 61, and 63) belong to these “bridges” and hence could not be assigned to any of the haplogroups—they were lumped into a haplogroup “I” (for indeterminate). We could then assign to each of the 75 strains a haplogroup genotype (Table 2). All strains belonging to the T. cruzi I lineage (MDS-cluster A in Figure 1) proved to be Z/Z (i.e., had two haplotypes belonging to haplogroup Z). Likewise, all the strains in MDS-cluster C (Figure 1) had Y/Y genotypes and those in MDS-cluster B had X/X genotypes. The strains in cluster BH all had X/Y genotypes confirming their hybrid nature. Strains Can III (genotype I/I, *COII* B), Dog Theis (genotype I/I, *COII* C), 402, and Mas1cl1 (both genotype I/Y, *COII* C), and M6241cl16 (genotype I/X, *COII* B) presented haplotypes of haplogroup I. It is noteworthy that three of these five strains are the ones outside MDS clusters in Figure 1A.

## Discussion

The population structure of T. cruzi is far from being completely understood. Although the existence of two major lineages in this species is well accepted, uncertainties about the existence or not of a third major ancestral group have been raised [15,35,36]. For instance, strains belonging to zymodeme Z3 or to rDNA group 1/2 could not be classified into either T. cruzi I or T. cruzi II [19]. Likewise, other strains (such as SC43) that present incongruities between the rDNA (group 2) and mini-exon (group 1) typing cannot be allocated into any of the two major lineages [24]. One of the major goals of this work was to investigate the genetic relationships among these “unclassifiable” strains.

Our first strategy was to perform the phylogenetic analysis of T. cruzi populations by using microsatellite data. Albeit extremely variable, these DNA markers allowed us to reliably identify four significant major clusters of strains (MDS clusters A, B, C, and BH in Figure 1). MDS-cluster A corresponds to *T cruzi* I and MDS-cluster C to classical T. cruzi II or T. cruzi IIb as named by Brisse et al. [26]. MDS-cluster B contains strains classified as Z3 and assigned to the IIc sublineage [26]. Finally, the strains within MDS-cluster BH were known to belong to the putative hybrid isozyme clonets 39 or 43 as proposed by Tibayrenc [43] and later classified as IId and IIe sublineages by Brisse et al. [26] (see Table 1).

Nucleotide sequencing and AluI RFLP analysis of a 290-bp stretch of the mitochondrial *COII* gene demonstrated that all strains enclosed in our microsatellite clusters B and BH (Z3 and hybrid strains) belonged to the same mitochondrial clade B. Sequences of two other mitochondrial genes, *CYb* [35] and *ND1* [37], obtained from GenBank, amply confirmed this observation by showing that indeed hybrid strains (sublineages IId and IIe) and Z3 strains (sublineage IIc) were grouped together into the same mitochondrial clade B. This same conclusion had been reached earlier [35,37].

Gaunt et al. [28] have shown that the hybridization of T. cruzi strains involves only nuclear genomes, without mitochondrial fusion. Here, we clearly demonstrated that the mitochondrial clade B is a third major phylogenetic division of T. cruzi, distinct from T. cruzi I (mitochondrial clade A) and T. cruzi II (mitochondrial clade C) major lineages. We have also shown that the strains with hybrid molecular markers in their nuclear genomes have a distinct mitochondrial genome (genotype B).

The analyses with all studied nuclear markers identified 141 different haplotypes that could be clustered into three haplogroups. All strains belonging to the T. cruzi I major lineage (MDS-cluster A in Figure 1) proved to be Z/Z (i.e., had two haplotypes belonging to haplogroup Z). Likewise, all the strains in MDS-cluster C (Figure 1) had Y/Y genotypes and those in MDS-cluster B had X/X genotypes. Thus, our data do not corroborate the suggestion made by Sturm et al. [36] that sublineage IIc (MDS-cluster B) is a hybrid. In contrast, the strains in MDS-cluster BH all had X/Y genotypes, confirming their hybrid character. Because of the way that PHASE identifies haplotypes, proximity of haplotype numbers is highly correlated with genetic proximity. Hybrid strains 167, 1022, 182, CLBrener, and Tulacl2 have, respectively, genotypes 4/99, 2/102, 5/108, 5/100, and 3/103, forming one group, while strains MNcl2, NR, SC43cl1, and SO3 have genotypes 52/133, 55/130, 54/129, and 54/130, and form another (notice equivalence with sublineages IIe and IId of Brisse et al. [26]). This indicates that at least two independent hybridizations occurred, presumably followed by clonal microdifferentiation.

**Figure 5:**
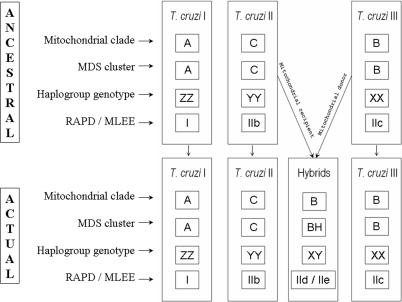
Diagram Depicting the Proposed Model for the Evolution of T. cruzi Strains The mitochondrial clade was typed by RFLP of the *COII* maxicircle gene, the MDS clusters were established by multidimensional scaling of microsatellite data, the haplogroups were established by haplotype estimation from multilocal genotypes followed by median joining network analysis, and the RAPD/multilocus enzyme electrophoresis typing was obtained from Brisse et al. [26].

Based on these results we propose the following minimal scenario for the evolution of T. cruzi populations (Figure 5). In the distant past there were at least three ancestral clades (MDS clusters A, C, and B in Figure 1) that we may call, respectively, T. cruzi I, T. cruzi II, and T. cruzi III. It is interesting to note that this proposal matches the initial suggestion made by Miles et al. [23] almost 30 years ago on the basis of isozyme studies. Most likely, T. cruzi II and T. cruzi III had overlapping ecological niches, and thus the conditions necessary for hybridization were in place. At least two hybridization events produced evolutionarily viable progeny. In both events, the cytoplasmic donor for the resulting offspring (as identified by the mitochondrial clade of the hybrid strains) was T. cruzi III. From the haplotype reconstitutions we can estimate the parentage of a hybrid strain. For instance, CLBrener, the reference strain for the recently completed T. cruzi genome sequencing [16], has genotype 5/100. Its most likely mitochondrial recipient was a strain proximate to 1005 (genotype 100/106), while the most likely mitochondrial donor was a close relative of strains 222 and 115, which are very near each other in Figure 1 (arrowheads). The existence of strains that cannot be accommodated into this scenario (i.e., CanIII [sublineage IIa of Brisse et al. [26]] and Dog Theis) indicates that the evolutionary history had additional complexities. However, our simple model (depicted in Figure 5) should be useful for proposing and testing evolutionary and pathogenetic hypotheses.

The fact that the same population structure of T. cruzi can be envisaged with different molecular markers, such as isozymes [23], RAPD [26,35], microsatellites [30], and several sequence-based nuclear [20,21,37,38] and mitochondrial ([35,37], this study) markers, bears witness to its extreme stability. Although, as shown conclusively in our study and also by others [35,37], hybridization events clearly did occur in the evolutionary history of *T. cruzi,* they seem to have been only occasional and to have been subsequently stabilized by strong clonal propagation (reviewed in [17,18]).

## Materials and Methods

### T. cruzi isolates.

T. cruzi stocks (75) isolated from both domestic and sylvatic transmission cycles were analyzed (Table 1). DNA from the parasites were kindly provided by Dr. Égler Chiari from the Departamento of Parasitologia, Universidade Federal de Minas Gerais (Belo Horizonte, Brazil); Dr. José Rodrigues Coura and Dr. Ana Maria Jansen-Franken, from the Departamento de Medicina Tropical and the Departamento de Protozoologia, Fundação Oswaldo Cruz (Rio de Janeiro, Brazil), respectively; and Dr. M. Tibayrenc from the Centre d'Études sur le Polymorphisme des Microorganismes (Montpellier, France).

### Nuclear genetic typing.

Amplification of five previously described microsatellite loci*,* denominated SCLE10, SCLE11, MCLE01, MCLF10, and MCLG10, was performed as previously described [30]. After the PCR, the amplified microsatellites were loaded on a 6% denaturing polyacrylamide gel and analyzed on an ALF sequencer (GE Healthcare, Milwaukee, Wisconsin, United States) using the Allelinks software (GE Healthcare). To determine the allele size the samples were directly compared with the band sizes from an allelic ladder prepared by amplification of an artificial mixture of DNA from 60 T. cruzi strains.

Amplification of the D7 divergent domain of the 24Sα rRNA gene was achieved by PCR with D71 fluorescent (5′-AAGGTGCGTCGACAGTGTGG-3′) and D72 (5′-TTTTCAGAATGGCCGAACAGT-3′) primers following protocols described previously [24]. The amplification products were also analyzed in ALF sequencer and allele sizes determined by the Allelinks software.

### Mitochondrial genetic typing.

Amplification of the mitochondrial *COII* gene [37] was performed using the primers TcMit31 (5′-TAAATAATATATATTGTACATGAG-3′) and TcMit40 (5′-CTRCATTGYCCATATATTGT-3′). Total DNA (1–10 ng) were used in each PCR reaction in the following condition: 30 s denaturation at 94 °, primer annealing for 2 min at 48 °, and primer extension for 2 min at 72 °, in a total of 30 cycles. The amplified products were purified and sequenced using primer TcMit31 and the cycle sequencing with Thermo-Sequenase (ETKit; GE Healthcare) using the thermal cycling program recommended in the kit. The sequencing products were purified and run on a MegaBACE capillary sequencer (GE Healthcare). After Phred, Phrap, and Consed analyses, the sequences were trimmed to have equal length (290 base pairs). All bases sequenced had Phred values above 30 [44].

Based on the restriction map of *COII* sequences, the AluI restriction endonuclease was chosen to perform RFLP analyses in the mitochondrial *COII* gene. After PCR amplification, the amplicons were submitted to enzyme digestion for 16 hours according to instructions provided by the manufacturer (Promega, Madison, Wisconsin, United States). Digested products were analyzed on polyacrylamide gel electrophoresis and silver stained.

Sequences for the mitochondrial *CYb* gene [35] and the *ND1* (37) were obtained from GenBank.

### Construction of distance matrices, multidimensional scaling, and NJ trees.

Based on the microsatellite results, a distance matrix between the strains was constructed as described previously [30]. In order to provide a visual representation of the distance matrix we used the multidimensional scaling plot using the software Statistica Version 6.0 [45]. Analyses of molecular variance for the mitochondrial sequences were performed using the Arlequin v.2.0 software using 1,000 permutations [46].

NJ trees were obtained separately for the *COII, CYb,* and *ND1* sequences with the MEGA v. 3.1 software [47] using the Kimura 2 parameter and 500 replications for the bootstrap statistics.

### Haplotype inference and network construction.

Haplotypes were reconstructed from the 75 T. cruzi populations by using a Bayesian coalescent theory-based method contained in PHASE software (Version 2.0.2 for Linux) [40]. The type of polymorphism (SNP or multiallelic with stepwise mutation mechanism for rDNA and microsatellite data, respectively) is taken into account in PHASE. For the analyses the default parameters of the program were used, with additional runs up to 10,000 permutations. These were the best-tested conditions, giving highly reproducible results. The resultant haplotypes were then arranged in a network by using the Median Joining analysis [41], available in NETWORK 3.1 software provided by Fluxus Technology (http://www.fluxus-engineering.com).

## Supporting Information

Table S1 Typing of rDNA Group and Allele Sizes (in bp) of Five Microsatellite Loci (105 KB DOC)

### Accession numbers

The GenBank (http://www.ncbi.nlm.nih.gov/Genbank) accession numbers for the genes and gene products discussed in this paper are 24S rDNA (L19411), mini-exon gene (X62674), *COII* (AF359041 and DQ343715–DQ343753), *CYb* (AJ130921, AJ130931–AJ130938, AJ439719–AJ439727), and *ND1* (AF359009, AF359011–AF359029, AF359031–AF359053).

## Abbreviations

*COII*: cytochrome oxidase subunit II
*CYb*: cytochrome b
MDS: multidimensional scaling plot
*ND1*: NADH dehydrogenase subunit 1
NJ: neighbor-joining
RAPD: random amplified polymorphic DNA
RFLP: restriction fragment-length polymorphism

## References

[ppat-0020024-b001] Prata A (2001). Clinical and epidemiological aspects of Chagas disease. Lancet Infect Dis.

[ppat-0020024-b002] World Health Organization [WHO] (1991). Control of Chagas disease. Technical Report Series, 811.

[ppat-0020024-b003] Briones MR, Souto RP, Stolf BS, Zingales B (1999). The evolution of two Trypanosoma cruzi subgroups inferred from rRNA genes can be correlated with the interchange of American mammalian faunas in the Cenozoic and has implications to pathogenicity and host specificity. Mol Biochem Parasitol.

[ppat-0020024-b004] Aufderheide AC, Salo W, Madden M, Streitz J, Buikstra J et al. (2004). A 9,000-year record of Chagas' disease. Proc Natl Acad Sci U S A.

[ppat-0020024-b005] Dias JC, Silveira AC, Schofield CJ (2002). The impact of Chagas disease control in Latin America: A review. Mem Inst Oswaldo Cruz.

[ppat-0020024-b006] Kirchhoff LV (1993). American Trypanosomiasis (Chagas' Disease)—a tropical disease now in the United States. N Engl J Med.

[ppat-0020024-b007] Jansen AM, Santos de Pinho AP, Lisboa CV, Cupolillo E, Mangia RH (1999). The sylvatic cycle of *Trypanosoma cruzi:* A still unsolved puzzle. Mem Inst Oswaldo Cruz.

[ppat-0020024-b008] Lisboa CV, Mangia RH, De Lima NR, Martins A, Dietz J (2004). Distinct patterns of Trypanosoma cruzi infection in Leontopithecus rosalia in distinct Atlantic coastal rainforest fragments in Rio de Janeiro–Brazil. Parasitology.

[ppat-0020024-b009] Pung OJ, Banks CW, Jones DN, Krissinger MW (1995). Trypanosoma cruzi in wild raccoons, opossums, and triatomine bugs in southeast Georgia, U.S.A. J Parasitol.

[ppat-0020024-b010] Pietrzak SM, Pung OJ (1998). Trypanosomiasis in raccoons from Georgia. J Wildl Dis.

[ppat-0020024-b011] Bradley KK, Bergman DK, Woods JP, Crutcher JM, Kirchhoff LV (2000). Prevalence of American trypanosomiasis (Chagas disease) among dogs in Oklahoma. J Am Vet Med Assoc.

[ppat-0020024-b012] Maguire JH, Hoff R, Sleigh AC, Mott KE, Ramos NB (1986). An outbreak of Chagas' disease in southwestern Bahia, Brazil. Am J Trop Med Hyg.

[ppat-0020024-b013] Shikanai-Yasuda MA, Marcondes CB, Guedes LA, Siqueira GS, Barone AA (1991). Possible oral transmission of acute Chagas' disease in Brazil. Rev Inst Med Trop Sao Paulo.

[ppat-0020024-b014] da Silva Valente SA, de Costa Valente V, Neto HF (1999). Considerations on the epidemiology and transmission of Chagas disease in the Brazilian Amazon. Mem Inst Oswaldo Cruz.

[ppat-0020024-b015] Pedroso A, Cupolillo E, Zingales B (2003). Evaluation of Trypanosoma cruzi hybrid stocks based on chromosomal size variation. Mol Biochem Parasitol.

[ppat-0020024-b016] El-Sayed NM, Myler PJ, Bartholomeu DC, Nilsson D, Aggarwal G (2005). The genome sequence of Trypanosoma cruzi, etiologic agent of Chagas disease. Science.

[ppat-0020024-b017] Macedo AM, Pena SDJ (1998). Genetic variability of *Trypanosoma cruzi:* Implications for the pathogenesis of Chagas' disease. Parasitol Today.

[ppat-0020024-b018] Tibayrenc M (2003). Genetic subdivisions within *Trypanosoma cruzi* (Discrete Typing Units) and their relevance for molecular epidemiology and experimental evolution. Kinetoplastid Biol Dis.

[ppat-0020024-b019] Satellite Meeting, (1999). Recommendations from an international symposium to commemorate the 90th anniversary of the discovery of Chagas disease. April 11–16 1999, Rio de Janeiro, Brazil. Mem Inst Oswaldo Cruz.

[ppat-0020024-b020] Fernandes O, Santos S, Junqueira A, Jansen A, Cupolillo E et al. (1999). Populational heterogeneity of Brazilian Trypanosoma cruzi isolates revealed by the mini-exon and ribosomal spacers. Mem Inst Oswaldo Cruz.

[ppat-0020024-b021] Zingales B, Stolf BS, Souto RP, Fernandes O, Briones MR (1999). Epidemiology, biochemistry and evolution of Trypanosoma cruzi lineages based on ribosomal RNA sequences. Mem Inst Oswaldo Cruz.

[ppat-0020024-b022] Freitas JM, Lages-Silva E, Crema E, Pena SDJ, Macedo AM (2005). Real time PCR strategy for the identification of major lineages of Trypanosoma cruzi directly in chronically infected human tissues. Int J Parasitol.

[ppat-0020024-b023] Miles MA, Souza A, Povoa M, Shaw JJ, Lainson R (1978). Isozymic heterogeneity of Trypanosoma cruzi in the first autochthonous patients with Chagas' disease in Amazonian Brazil. Nature.

[ppat-0020024-b024] Souto RP, Fernandes O, Macedo AM, Campbell DA, Zingales B (1996). DNA markers define two major phylogenetic lineages of Trypanosoma cruzi. Mol Biochem Parasitol.

[ppat-0020024-b025] Stolf BS, Souto RP, Pedroso A, Zingales B (2003). Two types of ribosomal RNA genes in hybrid Trypanosoma cruzi strains. Mol Biochem Parasitol.

[ppat-0020024-b026] Brisse S, Dujardin JC, Tibayrenc M (2000). Identification of six Trypanosoma cruzi phylogenetic lineages by random amplified polymorphic DNA and multilocus enzyme eletrophoresis. Int J Parasitol.

[ppat-0020024-b027] Macedo AM, Machado CR, Oliveira RP, Pena SDJ (2004). *Trypanosoma cruzi:* Genetic structure of populations and relevance of genetic variability to the pathogenesis of Chagas disease. Mem Inst Oswaldo Cruz.

[ppat-0020024-b028] Gaunt MW, Yeo M, Frame IA, Stothard JR, Carrasco H J, et al. (2003). Mechanism of genetic exchange in American trypanosomes. Nature.

[ppat-0020024-b029] Tibayrenc M, Ward P, Moya A, Ayala FJ (1986). Natural populations of *Trypanosoma cruzi,* the agent of Chagas' disease, have a complex multiclonal structure. Proc Nat Acad Sci U S A.

[ppat-0020024-b030] Oliveira RP, Broude NE, Macedo AM, Cantor CR, Smith CL et al. (1998). Probing the genetic population structure of Trypanosoma cruzi with polymorphic microsatellites. Proc Natl Acad Sci U S A.

[ppat-0020024-b031] Tibayrenc M, Ayala FJ (2002). The clonal theory of parasitic protozoa: 12 years on. Trends Parasitol.

[ppat-0020024-b032] Bogliolo AR, Lauria-Pires L, Gibson WC (1996). Polymorphisms in *Trypanosoma cruzi:* evidence of genetic recombination. Acta Trop.

[ppat-0020024-b033] Carrasco HJ, Frame IA, Valente SA, Miles MA (1996). Genetic exchange as a possible source of genomic diversity in sylvatic populations of Trypanosoma cruzi. Am J Trop Med Hyg.

[ppat-0020024-b034] Higo H, Yanagi T, Matta V, Agatsuma, Cruz-Reyes A (2000). Genetic structure of Trypanosoma cruzi in American continents: Special emphasis on sexual reproduction in Central America. Parasitology.

[ppat-0020024-b035] Brisse S, Henriksson J, Barnabe C, Douzery EJ, Berkvens D (2003). Evidence for genetic exchange and hybridization in Trypanosoma cruzi based on nucleotide sequences and molecular karyotype. Infect Genet Evol.

[ppat-0020024-b036] Sturm NR, Vargas NS, Westenberger SJ, Zingales B, Campbell DA (2003). Evidence for multiple hybrid groups in Trypanosoma cruzi. Int J Parasitol.

[ppat-0020024-b037] Machado CA, Ayala FJ (2001). Nucleotide sequences provide evidence of genetic exchange among distantly related lineages of Trypanosoma cruzi. Proc Natl Acad Sci U S A.

[ppat-0020024-b038] Augusto-Pinto L, Teixeira SM, Pena SDJ, Machado CR (2003). Single-nucleotide polymorphisms of the *Trypanosoma cruzi MSH2* gene support the existence of three phylogenetic lineages presenting differences in mismatch-repair efficiency. Genetics.

[ppat-0020024-b039] Excoffier L, Smouse PE, Quattro JM (1992). Analysis of molecular variance inferred from metric distances among DNA haplotypes: Application to human mitochondrial DNA restriction data. Genetics.

[ppat-0020024-b040] Stephens M, Smith NJ, Donnelly P (2001). A new statistical method for haplotype reconstruction from population data. Am J Hum Genet.

[ppat-0020024-b041] Bandelt HK, Forster P, Rohl A (1999). Median-joining networks for inferring intraspecific phylogenies. Mol Biol Evol.

[ppat-0020024-b042] Leopoldino AM, Pena SDJ (2003). The mutational spectrum of human autosomal tetranucleotide microsatellites. Hum Mutat.

[ppat-0020024-b043] Tibayrenc M (1996). Towards a unified evolutionary genetics of microorganisms. Annu Rev Microbiol.

[ppat-0020024-b044] Ewing B, Hillier L, Wendl MC, Green P (1998). Base-calling of automated sequencer traces using phred. I. Accuracy assessment. Genome Res.

[ppat-0020024-b045] Beals R, Krantz DH, Tversky A (1968). Foundations of multidimensional scaling. Psychol Rev.

[ppat-0020024-b046] Schneider S, Roessli D, Excoffier L (2000). Arlequin ver 2.000: A software for population genetics data analysis [computer program].

[ppat-0020024-b047] Kumar S, Tamura K, Nei M (2004). MEGA3: Integrated software for molecular evolutionary genetics analysis and sequence alignment. Briefings Bioinform.

