# Supplementary material for: Ancestral Genomes, Sex, and the Population Structure of Trypanosoma cruzi
Source: PLoS Pathog. 2006 Mar 31;2(3):e24. doi: 10.1371/journal.ppat.0020024 (PMC1434789; doi:10.1371/journal.ppat.0020024)
Supplement: Table S1 — (105 KB DOC) [file ppat.0020024.st001.doc]

**Supporting information.**

**Table S1: Typing of rDNA group and allele sizes (in bp) of five microsatellite loci**

| Strains | RDNA  group | SCLE10 | SCLE11 | MCLE01 | MCLF10 | MCLG10 |
| --- | --- | --- | --- | --- | --- | --- |
| 84 | 1 | 267/271 | 153/155 | 126/134 | 182/182 | 177/177 |
| 115 | 1\2 | 245/251 | 153/153 | 132/132 | 182/190 | 177/177 |
| 167 | 1 | 237/275 | 153/153 | 130/130 | 182/194 | 177/177 |
| 182 | 1 | 237/275 | 153/157 | 130/146 | 182/194 | 177/177 |
| 207 | 1 | 245/251 | 151/155 | 134/142 | 190/190 | 177/177 |
| 209 | 1 | 269/275 | 153/155 | 132/132 | 182/182 | 177/177 |
| 222 | 2 | 215/251 | 151/155 | 132/132 | 190/190 | 177/177 |
| 226 | 1\2 | 245/251 | 153/155 | 132/132 | 182/184 | 177/177 |
| 231 | 2 | 245/251 | 151/155 | 134/134 | 182/194 | 175/175 |
| 239 | 1 | 271/275 | 151/157 | 134/150 | 182/182 | 177/177 |
| 402 | 2 | 271/255 | 139/139 | 138/144 | 182/182 | 175/175 |
| 577 | 1 | 273/281 | 151/155 | 152/154 | 186/186 | 173/177 |
| 578 | 1 | 281/281 | 149/155 | 130/148 | 186/186 | 177/177 |
| 580 | 1 | 279/279 | 149/149 | 130/134 | 184/184 | 177/177 |
| 581 | 1 | 277/277 | 153/161 | 130/148 | 186/186 | 175/175 |
| 803 | 1 | 271/281 | 145/145 | 136/152 | 184/184 | 177/183 |
| 1001 | 2 | 255/255 | 143/143 | 130/130 | 182/182 | 149/153 |
| 1004 | 2 | 253/255 | 139/139 | 130/130 | 186/190 | 155/155 |
| 1005 | 1 | 275/275 | 153/155 | 130/156 | 182/182 | 177/179 |
| 1006 | 2 | 253/255 | 139/141 | 130/130 | 184/188 | 153/153 |
| 1014 | 1 | 269/275 | 149/149 | 140/142 | 182/194 | 177/177 |
| 1022 | 1 | 233/275 | 153/153 | 130/134 | 182/194 | 177/177 |
| 1043 | 1 | 275/281 | 157/157 | 128/134 | 180/180 | 177/179 |
| 1502 | 2 | 255/255 | 141/143 | 136/136 | 186/186 | 153/153 |
| 1523 | 2 | 255/255 | 143/147 | 144/144 | 180/188 | 153/153 |
| 1931 | 1 | 275/275 | 141/153 | 140/140 | 184/184 | 181/181 |
| 3663 | 2 | 245/245 | 155/155 | 128/132 | 184/194 | 175/175 |
| 3869 | 2 | 249/249 | 153/157 | 132/132 | 186/192 | 175/179 |
| 4182 | 2 | 249/263 | 155/159 | 126/128 | 196/200 | 175/175 |
| 183744 | 1 | 281/281 | 149/149 | 130/134 | 184/184 | 177/177 |
| 169/1 | 1 | 273/275 | 149/149 | 128/138 | 180/184 | 177/177 |
| 200pm | 1 | 277/277 | 153/155 | 136/136 | 184/184 | 177/179 |
| 84Ti | 1 | 271/271 | 143/147 | 136/136 | 184/184 | 177/185 |
| A83 | 2 | 253/253 | 141/141 | 134/138 | 186/186 | 155/155 |
| A87 | 2 | 253/253 | 141/141 | 142/150 | 186/186 | 153/155 |
| Be62 | 1 | 273/275 | 155/155 | 136/138 | 182/182 | 177/177 |
| CanIII cl1 | 119** | 259/259 | 139/139 | 130/130 | 190/190 | 155/173 |
| CLBrener | 1 | 237/275 | 153/153 | 130/130 | 182/182 | 177/177 |
| Col18/05 | 2 | 253/257 | 139/139 | 136/136 | 182/184 | 155/155 |
| Colombiana | 2 | 235/257 | 139/139 | 136/136 | 184/186 | 157/157 |
| CPI11/94 | 1 | 277/297 | 153/153 | 130/130 | 184/184 | 177/177 |
| CPI95/94 | 1 | 277/277 | 149/155 | 124/132 | 184/184 | 177/177 |
| Cuíca | 2 | 253/255 | no amplif. | 130/130 | 192/192 | 155/155 |
| Cutia | 2 | 255/255 | 143/143 | 130/130 | 186/190 | 155/155 |
| D7 | 2 | 245/253 | 143/143 | 134/142 | 186/186 | 155/155 |
| Dog Theis | 1 | 261/261 | 141/151 | 136/136 | 184/184 | 173/175 |
| Esmeraldo | 1 | 275/287 | 151/159 | 124/136 | 180/182 | 177/179 |
| Gamba cl1 | 2 | 253/255 | 143/143 | 130/130 | 188/188 | 155/155 |
| Gil | 1 | 271/271 | 155/157 | 136/144 | 180/182 | 175/175 |
| GLT564 | 1 | 275/275 | 151/151 | 136/136 | 182/182 | 177/181 |
| GLT593 | 1 | 271/285 | 151/151 | 136/136 | 180/180 | 179/183 |
| GMS | 1 | 273/285 | 149/157 | 126/134 | 182/182 | 177/177 |
| GOCH | 1 | 271/281 | 153/159 | 128/150 | 186/186 | 173/173 |
| Ig539 | 1 | 281/281 | 151/153 | 132/150 | 184/184 | 173/179 |
| JAF | 1 | 269/275 | 143/149 | 130/134 | 182/182 | 177/179 |
| JG | 1 | 273/275 | 145/149 | 134/136 | 182/182 | 177/179 |
| JHF | 1 | 273/273 | 149/149 | 134/136 | 180/180 | 175/179 |
| JSM | 1 | 267/275 | 143/151 | 128/128 | 182/182 | 181/181 |
| M5631cl5 | 2 | 247/247 | 155/155 | 134/134 | 190/190 | 175/175 |
| M6241cl6 | 2 | 253/253 | 151/153 | 128/130 | 182/194 | 177/177 |
| Mas1 cl1 | 1 | 265/287 | 151/151 | 128/128 | 184/184 | 177/177 |
| MN cl2 | 1\2 | 255/287 | 149/149 | 130/130 | 182/192 | 177/179 |
| MPD | 1 | 267/271 | 143/153 | 138/138 | 180/180 | 177/181 |
| NR | 1\2 | 255/285 | 149/165 | 128/130 | 182/190 | 177/177 |
| OPS27/94 | 1 | 287/297 | 149/159 | 132/136 | 184/184 | 177/177 |
| Rb1 | 2 | 255/259 | 139/139 | 128/136 | 188/188 | 153/155 |
| Rb2 | 2 | 251/255 | 139/141 | 136/142 | 180/188 | 153/155 |
| Rb6 | 2 | 255/255 | no amplif. | 132/134 | 180/186 | 153/153 |
| SC43 cl1 | 2 | 255/285 | 149/163 | 128/130 | 182/190 | 177/177 |
| SE | 2 | 287/297 | 143/151 | 136/140 | 180/190 | 153/153 |
| SilvioX10 cl1 | 2 | 235/275 | 153/153 | 130/130 | 184/196 | 177/177 |
| SO3 | 2 | 253/255 | 143/143 | 132/132 | 192/192 | 155/155 |
| Tu18 cl11 | 1\2 | 255/285 | 149/163 | 128/130 | 182/190 | 177/177 |
| Tula cl2 | 1 | 289/289 | 149/151 | 130/130 | 182/182 | 177/177 |
| Y | 1 | 273/273 | 153/153 | 130/130 | 182/184 | 177/177 |

** = rDNA allele size of 119 bp
